# A Signaling Protease Required for Melanization in *Drosophila* Affects Resistance and Tolerance of Infections

**DOI:** 10.1371/journal.pbio.0060305

**Published:** 2008-12-09

**Authors:** Janelle S Ayres, David S Schneider

**Affiliations:** Department of Microbiology and Immunology, Stanford University, Stanford, California, United States of America; University of Georgia, United States of America

## Abstract

Organisms evolve two routes to surviving infections—they can resist pathogen growth (resistance) and they can endure the pathogenesis of infection (tolerance). The sum of these two properties together defines the defensive capabilities of the host. Typically, studies of animal defenses focus on either understanding resistance or, to a lesser extent, tolerance mechanisms, thus providing little understanding of the relationship between these two mechanisms. We suggest there are nine possible pairwise permutations of these traits, assuming they can increase, decrease, or remain unchanged in an independent manner. Here we show that by making a single mutation in the gene encoding a protease, CG3066, active in the melanization cascade in Drosophila melanogaster, we observe the full spectrum of changes; these mutant flies show increases and decreases in their resistance and tolerance properties when challenged with a variety of pathogens. This result implicates melanization in fighting microbial infections and shows that an immune response can affect both resistance and tolerance to infections in microbe-dependent ways. The fly is often described as having an unsophisticated and stereotypical immune response where single mutations cause simple binary changes in immunity. We report a level of complexity in the fly's immune response that has strong ecological implications. We suggest that immune responses are highly tuned by evolution, since selection for defenses that alter resistance against one pathogen may change both resistance and tolerance to other pathogens.

## Author Summary

To boost its defenses, an organism may increase its resistance to infection by reducing the fitness of the invading pathogen; alternatively, the host may increase its tolerance by reducing the damage caused by a given quantity of pathogen. Melanization is an immune response that has been linked to defense in the fly and other invertebrates. It is expected to cause resistance to infection, as well as host damage mediated by reactive oxygen species generated during melanin production. We demonstrate here that the loss of a gene required for melanization produces a surprisingly complex spectrum of phenotypes, increasing and decreasing both resistance and tolerance to a variety of microbes. For example, increasing resistance to one pathogen can produce corresponding changes in either resistance or tolerance to another pathogen. As a result, there is likely no “best” solution that produces a perfect immune system, only an equilibrium that allows the fly to deal with the pathogenic threats that its ancestors have faced. This equilibrium will require the balancing of both resistance and tolerance, and our study demonstrates that we cannot completely understand the defensive properties of a host unless we measure both of these properties in response to a variety of pathogens.

## Abstract 3

It isn't easy to predict how mutations will affect a host's immune response. Mutations that affect the interaction of a fly with one pathogen can increase or decrease resistance or tolerance to other pathogens.

## Introduction

Evolutionary theory suggests that a host can protect itself against an infectious pathological threat by evolving two different mechanisms to increase fitness or health. The first is to reduce the fitness of the pathogen, thereby reducing the number of pathogens attacking the host. The second is to limit the health costs to the host. The sum of both these mechanisms defines an individual host's defensive capabilities. In the plant ecology community, these two mechanisms are defined as resistance and tolerance [1–5]. Dividing the physiological response to infections into these two components is important because it demonstrates that the health of a host cannot be measured solely by its ability to survive an infection and that studying both pathogen clearance and pathology is essential to fully understanding the defensive measures of a host. Typically in animal immunity studies we focus on understanding resistance mechanisms. For example, most work on *Drosophila* immunity to date has concentrated on pattern recognition pathways that, when mutated, permit overgrowth of bacteria and thus reduce host defenses [6–12]. There is evidence that tolerance properties exist in *Drosophila*, but the relationship between these mechanisms and resistance mechanisms, as well as their effects on host defense, have not been examined [13–17].

Tolerance as defined by the evolutionary community measures the slope of fitness versus pathogen load [18–20]. These parameters are quite difficult to assay in *Drosophila* infections; in the fly, morbidity is most easily measured by measuring the mean time to death and therefore we do not, strictly speaking, assay fitness. In addition, it is difficult to measure the number of infecting bacteria in a fly without killing the fly, and thus we cannot easily relate the bacterial number in a given fly with mortality because both assays are destructive. We define tolerance in the fly system by stating that a fly that can survive a given level of microbes better than another fly is better able to tolerate an infection.

Changes in tolerance and resistance could have complicated effects on host defenses. We predict that for any given mutation, there are nine qualitatively different potential ways of affecting resistance and tolerance of a host, though the actual number of states is infinite (Figure 1). We imagine that both properties could be enhanced, diminished, or left unchanged by a single mutation. In our fly infection system not all nine of these combinations will be readily distinguishable. We inject flies with a pathogen and then monitor host survival and bacterial growth. Changes in resistance in mutants are detected by measuring pathogen growth and comparing these levels to those observed in infected wild-type flies. Tolerance of mutant flies is measured functionally as a change in survival when pathogen levels resemble that of infected wild-type flies. This mode of measurement prevents us from measuring changes in tolerance when microbe levels are also changing. Therefore, we predict that we should be able to differentiate among only five of these nine classes unless there are special circumstances, as described below.

**Figure 1:**
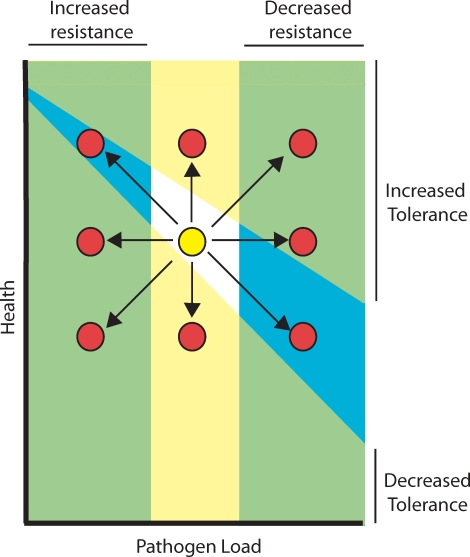
Manipulation of Resistance and Tolerance Affects Host Health We hypothesize that resistance and tolerance of a host can be manipulated in an independent manner, generating nine possible pairwise permutations to affect overall host health. Mutant phenotypes can be mapped onto a two-dimensional space where the axes are defined by health and pathogen load. We measure median survival time as a proxy for health and measure bacterial load directly in homogenized flies. The red dot represents the phenotype of a wild-type fly strain infected with a pathogen. Any given mutation could either have no effect or shift the phenotype to any of the eight red dots. Theoretically, phenotypic shifts can occur by altering either the resistance of the host, the tolerance, or both properties. The areas marked in blue show the area where shifts in resistance are expected to move the phenotypes; the pale yellow bar indicates the areas affected by shifts in tolerance, and the green shows areas that are caused by changes in both properties.

To test this prediction we examined the effects of altering the melanization arm of the *Drosophila* immune response on fly defense to a variety of pathogens. We chose this immune response because we anticipated that it not only functioned as a resistance mechanism in the fly and directly fights infections but could also cause considerable pathology in the host because it generates reactive oxygen; we expected this pathology would lead to changes in tolerance. Melanization is a presumed immune mechanism in the fly that produces melanin, visible as dark brown deposits, at the site of wounds and infection. Melanin is deposited after a chain of events induced by pattern recognition proteins, propagated by serine proteases and ultimately produced by the enzyme phenoloxidase [21–23]. In *Drosophila*, one such serine protease is CG3066, which acts in a melanization cascade that is negatively regulated by the serpin Spn27A [24,25]. Conventional wisdom suggests that melanin can sequester microbes to prevent their spread and that reactive oxygen species generated during melanin production can be directly harmful to microbes and possibly the host. There is evidence from work in other invertebrates, such as the crayfish Pacifastacus leniusculus, demonstrating that PPO activity is important for limiting microbial virulence [26–29]; however, the available literature concludes that melanization in *Drosophila* plays no role in fighting microbial infections, or it plays a redundant role, at best [24,25,30]. Though quite well defined biochemically, the functional contribution of this potential effector pathway to immunity remains in dispute.

In the present study we show that by making a single mutation in the melanization arm, specifically CG3066, of the fly innate immune response, we could alter both tolerance and resistance in a microbe dependent fashion. By doing so we observed five of the nine predicted phenotypic classes for changes in resistance and tolerance. Among these five we found two cryptic phenotypes in which there was no change in survival of the mutant flies but bacterial levels were very different from those found in wild-type flies. This suggests that resistance and tolerance had achieved a new balance in these flies. We also found an unanticipated phenotype of *CG3066* flies; these flies die significantly faster than wild-type flies when injected with sterile medium. We suggest that, in addition to its effects on the outcome of infections, this protease is important for tolerating some of the pathology that occurs during wounding. Typically in fly immunity, mutations have been reported to produce only two phenotypic classes—the flies either become sensitive to infections or their phenotype is unchanged. This work shows a level of complexity that has been missing in the description of *Drosophila* immunity. We suggest that these results have important implications about the evolution of immunity and that the equilibrium between resistance and tolerance of a host will be optimized by its interactions with pathogens in the wild.

## Results

### Some Bacterial Infections Elicit a Disseminated Melanization Response in the Fly

Previous studies examining the contribution of melanization to fly immunity did not test microbes that induce large melanization responses in the fly. Tang et al. observed that flies pierced with a needle containing a mixture of E. coli and Micrococcus luteus caused melanization at the site of needle insertion and concluded that this response was specific to the infection [25]. Although it is possible that the melanization observed was triggered by the infection, this cannot be concluded with confidence because injection with a sterile needle also results in deposits of melanin at the site of wounding approximately 24 h postinjection. Leclerc et al. did not report observations of melanization [24]. In Listeria monocytogenes– and Salmonella typhimurium–infected flies we observed, in addition to melanization at the site of injection, deposits of melanin just underneath the cuticle as well as in deeper tissues. This melanization is easily seen approximately 4 d (L. monocytogenes) or 7 d (S. typhimurium) after infection (Figure 2; unpublished data). We refer to this as a disseminated melanization response. We were curious if other bacteria elicited disseminated melanization during infection. We selected a diverse panel of bacteria and compared the patterns of melanization observed with media-injected control flies (Table 1).

**Figure 2:**
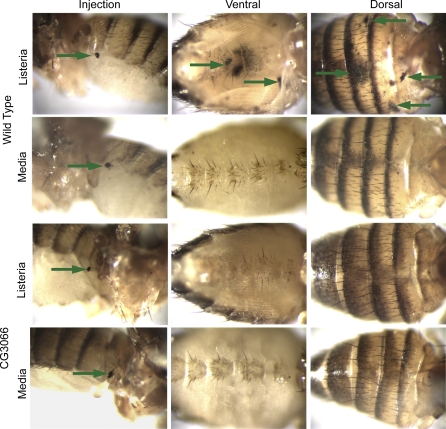
Disseminated Melanization in Infected Flies Ventral, dorsal, and injection site views of the abdomen of L. monocytogenes– or medium-injected female *w^1118^* and *CG3066* mutants. Deposits of melanin at the injection site (green arrows) can be seen in both wild-type and mutant flies for both microbe and medium injections. No melanin is observed on either fly strain beyond what is seen at the injection site in the absence of infection. Large amounts of melanin are deposited on both the ventral and dorsal sides of the abdomen in wild-type flies when infected with L. monocytogenes, while no melanin is observed in L. monocytogenes-infected *CG3066* mutants.

**Table 1:**
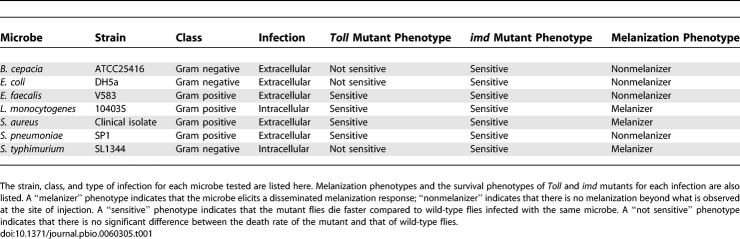
Microbial Strain List

Within the first 24 h postinfection, we saw melanin at the site of injection that was comparable to what we observed in flies that received a control injection of media. This was true for all bacteria tested. In addition to the melanization at the injection site, we found that L. monocytogenes, S. typhimurium, and Staphylococcus aureus all elicit a robust disseminated melanization response in infected flies. On average we found that more than 90% of females and more than 70% of males infected with L. monocytogenes exhibit disseminated melanization, and the majority of these flies have spots of melanin deposited along the dorsal and ventral sides of the abdomen (Figures 2 and 3). These deposits can be found on the cuticle of both sexes, and large melanin clots are commonly observed within the ovaries of females. To a lesser extent we also find melanization along the thorax and the head. In S. typhimurium–infected flies, on average, more than 80% of females and 70% of males exhibit a disseminated melanization response over the course of the infection, and we observe similar patterns of melanization to what we see with L. monocytogenes. The majority of flies exhibit melanization in the abdomen on the cuticle and also in the ovaries of females (Figure 2). In contrast to L. monocytogenes infections, we did not see melanization in the thorax or the head segments with S. typhimurium. S. aureus–infected flies exhibit a different pattern of melanization; we found approximately 40%–50% of both infected females and males exhibit a punctuate patterning of melanin deposits localized to the dorsal vessel. On occasion there are large melanized particles deeper in the tissue of the abdomen. No melanin is deposited along the thorax or the head. We did not observe melanization beyond that seen at the injection site in flies infected with the remaining bacteria tested: Enterococcus faecalis, Streptococcus pneumoniae, Escherichia coli, and Burkholderia cepacia (Figure 3).

**Figure 3:**
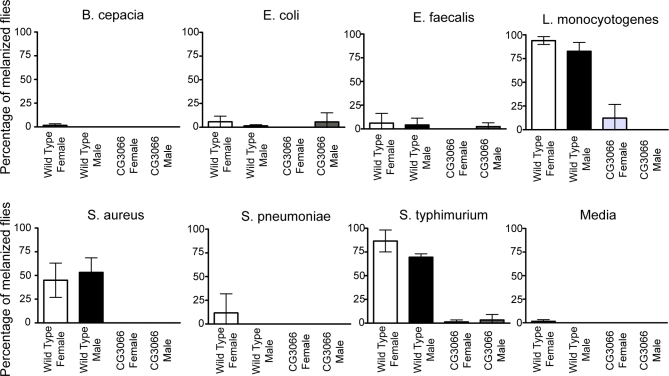
Quantification of Disseminated Melanization in Infected Flies Male and female 5- to 7-d-old *w^1118^* and *CG3066* mutants were infected with the microbes listed in Table 1 or with medium and examined for disseminated melanization throughout the course of infections with L. monocytogenes, S. typhimurium, S. aureus, E. faecalis, S. pneumoniae, E. coli, and *B. cepacia*. Melanization can be observed in L. monocytogenes-infected flies approximately 4 d postinfection, while melanization in S. typhimurium and S. aureus-infected flies can be seen approximately 7 d postinfection. Values indicate the percentage of infected wild-type or *CG3066* mutants that exhibit disseminated melanization, and data are represented as mean ± standard error of the mean. At least three groups of 20 flies were examined for each condition, and experiments were repeated three times and yielded similar results.

### Absence of Phenol Oxidase Activation Alters *Drosophila* Resistance and Tolerance Properties

Once we identified bacteria that elicit a disseminated melanization response we wanted to test whether this melanization response was important for a fly's survival and how this response affects resistance and tolerance during an infection. There are three genes encoding phenoloxidases in the fly, monophenoloxidase (Bc), diphenol oxidase a2, and diphenol oxidase a3 [31]. The *Bc* gene has received the most attention for its involvement in immunity because of its single characterized mutant, which eliminates circulating phenoloxidase from the hemolymph [32–34]. This allele *Bc* ^1^ is assumed to map to the monophenol oxidase gene; however, its molecular nature has not been reported [35]. *Bc* ^1^ is a dominant mutation that appears to prematurely activate phenoloxidase in larval crystal cells. A troubling aspect of this mutation is that it damages crystal cells and causes them to be phagocytosed by plasmatocytes, and the melanized remains of these cells sit undigested in the hemocytes for the life of the fly [36]. We anticipated that this *Bc* mutation could have pleiotropic effects on the immune response; it blocks phenoloxidase activity, but it is also anticipated to alter the cellular immune response because it destroys one immune cell outright and causes another to be filled with undigestible material. Since the cellular immune response plays an important role in fighting many infections, we sought another way to reduce melanization.

Leclerc et al. identified the protease encoded by *CG3066* as a prophenoloxidase activating enzyme (PPO), whereas Tang et al. reported that CG3066 enzyme was required for PPO activation but did not directly target PPO. A mutation of this gene was reported to inhibit the immune induced proteolytic cleavage of a *Drosophila* protein that cross-reacted with a mosquito anti-phenoloxidase antibody [24]. RNAi inhibition of this gene blocked the induction of phenoloxidase activity in fly extracts [25]. Thus *CG3066* mutants appeared to be a useful tool for dissecting the role melanization might play in resistance and tolerance. We found that these mutants are capable of producing melanin deposits at the site of injection for both media and microbial challenges comparable to that observed in wild-type flies (Figure 2); however, we did not observe a disseminated response in the *CG3066* mutants with L. monocytogenes, S. typhimurium, S. aureus, or any of the other bacteria tested (Figures 2 and 3).

To determine how CG3066 affects both tolerance and resistance properties of *Drosophila* we challenged *CG3066* mutant flies with our panel of bacteria and measured survival rates and bacterial loads (Figures 4 and 5). The microbes we tested produced infections that fell into five different phenotypic classes.

**Figure 4:**
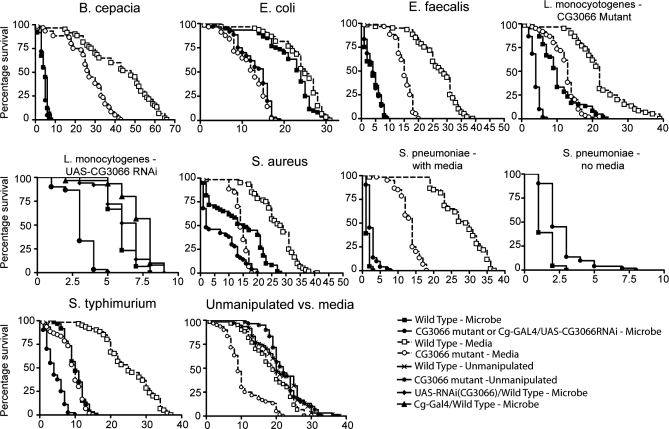
Survival of Infected *CG3066* Mutants Male 5- to 7-d-old flies were infected with bacteria or medium and survival was recorded daily for B. cepacia; E. coli; E. faecalis; L. monocytogenes infection in *CG3066^K02818^* mutant (*p* < 0.0001); L. monocytogenes infection in UAS-CG3066 RNAi (*p* < 0.0001); S. aureus (*p* < 0.0001) and S. pneumoniae (*p* < 0.0001) with medium control; S. pneumoniae without medium control (*p* < 0.0001); S. typhimurium (*p* < 0.0001) and medium versus unmanipulated flies (*p* < 0.0001). Filled squares represent microbe-injected wild-type flies, filled circles indicate microbe-injected *CG3066* mutant flies, open squares/dashed lines represent medium-injected wild-type flies, open circles/dashed lines indicate medium-injected *CG3066* mutants, × indicates unmanipulated wild-type flies, and asterisks (*) indicate unmanipulated *CG3066* mutants. For RNAi experiments expression was driven by the collagen-GAL4 driver, Cg-GAL4. Squares represent wild-type flies, circles represent Cg-GAL4/UAS-CG3066 RNAi, triangles represent Cg-GAL4/wild type, and diamonds represent UAS-CG3066 RNAi/wild type. Data are plotted as Kaplan-Meier plots and statistical significance was determined using log-rank analysis. Survival curves were repeated at least three times and yielded similar results each time.

**Figure 5:**
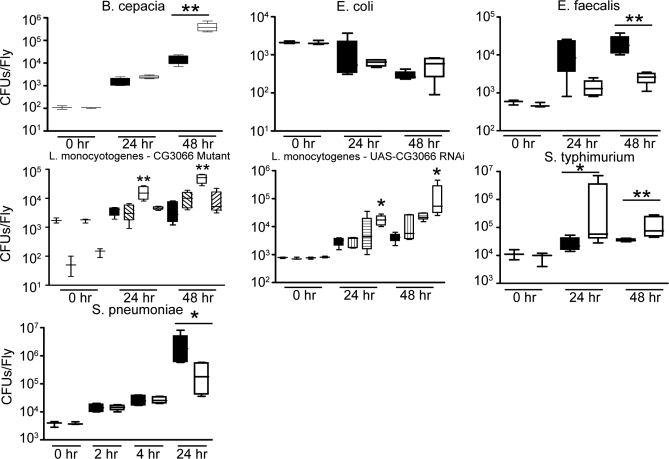
Bacterial Growth in *CG3066* Mutants Bacterial growth was determined for *B. cepacia; E. coli; E. faecelis*; L. monocytogenes growth in *CG3066^K02818^* mutants; L. monocytogenes growth in UAS-CG3066 RNAi; and E. coli, and S. pneumoniae. Male 5- to 7-d-old flies were infected with bacteria and collected at 0, 24, and 48 h postinfection. S. pneumoniae–infected flies were collected at 0, 2, 4, and 24 h postinfection. Flies were homogenized at each time point, serially diluted, and plated. For L. monocytogenes infection, flies were injected with 50 nl of 1 mg/ml gentamicin or water 3 h prior to plating. For B. cepacia, E. coli, S. pneumoniae, and S. typhimurium, solid black boxes indicate wild type, and solid white boxes indicate *CG3066* mutant. For gentamicin chase of L. monocytogenes-infected *CG3066* mutants, solid black boxes indicate water-treated wild-type flies, solid white boxes indicate water-treated *CG3066* mutants, left diagonal hashed boxes indicate gentamicin-treated wild-type flies, and right diagonal hashed boxes indicate gentamicin-treated *CG3066* mutants. For L. monocytogenes infection in RNAi flies, solid black boxes indicate wild-type flies, solid white boxes indicate Cg-GAL4/UAS-CG3066 RNAi, horizontal hashed boxes indicate Cg-GAL4/wild type, and vertical lined boxes indicate UAS-CG3066 RNAi/wild type. The *p*-value was determined with a nonparametric two-tailed *t*-test. Experiments for each microbe were repeated at least three times and gave similar results. * *p* < 0.01; ** *p* < 0.005.

The first class includes L. monocytogenes and *S. typhimurium.* These microbes killed *CG3066* mutants faster than wild-type flies and showed increased bacterial growth rates. S. typhimurium–infected mutants exhibited a 60% reduction in the median time to death (*p* < 0.0001) with respect to wild-type flies, and there was a 50% reduction in survival in *L. monocytogenes*–infected flies (*p* < 0.0001) (Figure 4). Using the UAS-GAL4 system and transgenic flies expressing double-stranded RNA targeting *CG3066*, we confirmed this reduction in survival by RNAi (*p* < 0.0001) (Figure 4). S. typhimurium and L. monocytogenes grew to significantly higher levels at both 24 and 48 h postinfection in *CG3066* mutants as compared to isogenic, wild-type parental controls (for both L. monocytogenes and S. typhimurium at 24 h *p* < 0.05, for both L. monocytogenes and S. typhimurium at 48 h *p* < 0.005) (Figure 5). This demonstrates that CG3066 plays an important and primary role in fighting some bacterial infections in the fly.

L. monocytogenes establishes an intracellular infection in wild-type *Drosophila*. We performed a gentamicin chase experiment to determine the location of the L. monocytogenes in mutant flies. Following infection, flies were injected with gentamicin, which will kill extracellular bacteria, while intracellular bacteria are protected from the antibiotic [16]. Control flies were injected with medium. Following a 3-h chase, flies were homogenized and plated to determine bacteria levels. This allowed us to measure the numbers of both intracellular and extracellular bacteria in the fly and to determine the contribution this protease might have on both populations of bacteria. We found significantly more bacteria in the *CG3066* mutant flies that received the medium chase compared to those that received the gentamicin chase, suggesting that there is an extracellular population of L. monocytogenes present in these mutants that is not present in wild-type flies (24 h, *p* = 0.0022; 48 h, *p* = 0.0043) (Figure 5). Similarly, we found an increase in L. monocytogenes growth when CG3066 expression is knocked down using RNAi (Figure 5). We conclude that CG3066 is normally important in controlling the growth of these microbes by enhancing the resistance properties of the fly, and this is similar to the sort of phenotype that has been seen for most *Drosophila* immunity mutants.

The second class of microbes we found is defined by E. coli, which is a nonmelanizer and showed no change in killing rates or bacterial levels in *CG3066* mutants. We define pathogenic bacteria as those that cause disease in wild-type flies; using this criterion, E. coli was the only nonpathogenic microbe we tested. An E. coli infection does not kill wild-type flies any faster than control flies injected with medium. We saw the same result in *CG3066* homozygous mutant flies; E. coli infected mutants die at the same rate as medium injected mutants (Figure 4). Colony counts in infected *CG3066* mutant flies were the same as seen in wild-type flies with an E. coli infection (Figure 5). This indicates that CG3066 has no effects on either fly resistance or tolerance with this type of infection.

S. pneumoniae defines our third class of microbes; *CG3066* mutants die significantly slower when infected with S. pneumoniae compared to wild-type flies. The median time to death in *CG3066* mutants was extended by 100% (*p* < 0.0001) (Figure 4). This increase in survival could have been due to changes in either resistance or tolerance. If resistance was altered, we anticipated that there would be differences in the levels of S. pneumoniae in the mutant flies, while changes in tolerance would leave the bacterial levels constant. We found that S. pneumoniae grew at a slower rate in *CG3066* mutants than in wild-type flies, leading us to conclude that *CG3066* mutant flies have better resistance against S. pneumoniae infection when melanization is absent. Alternatively, the presence of a functional melanization response could actually promote an S. pneumoniae infection in some manner. These results were surprising because we anticipated that the removal of a resistance response might increase the tolerance of the fly, but did not anticipate that it would increase the resistance of the host.

The fourth and fifth classes are cryptic and are defined by E. faecalis and B. cepacia, respectively. Our results with E. faecalis were in some ways similar to what has been published previously; we found that E. faecalis killed wild-type and *CG3066* mutant flies at the same rate. This result led Leclerc et al. to the conclusion that this mutation has no net effect on immunity [24]; however, we found that colony counts of the infected flies demonstrated that the story is more complicated than survival rates alone would lead us to believe. Infected *CG3066* mutant flies had significantly lower levels of E. faecalis than do wild-type flies at 48 h postinfection (Figure 5). This result suggests that the resistance properties of these flies are increased with respect to E. faecalis because the fly is better able to kill this type of bacteria. Given that the survival rates of these flies is the same as that of wild-type flies, this increase in resistance properties appears to be balanced by a reduction in tolerance. We conclude that a lower number of E. faecalis can cause disease symptoms in *CG3066* flies.

In contrast to what we observed during E. faecalis infections, we found that although B. cepacia kills wild-type and *CG3066* mutant flies at the same rate, there is increased growth of B. cepacia in the mutant flies. B. cepacia–infected flies exhibit a median time to death of 5 d post-infection (Figure 4). By 48 h postinfection, we find there is approximately 25 times more bacteria in the mutant flies compared to wild-type flies (*p* = 0.0043) (Figure 5). This increased bacterial growth suggests that the resistance mechanisms in the mutant flies are less effective at fighting a B. cepacia infection. Because this increased bacterial growth is coupled with no change in survival we suggest that the decrease in resistance properties is accompanied by an increase in tolerance, which is the opposite of what we see with an E. faecalis infection. These E. faecalis and B. cepacia experiments highlight the importance of using multiple tests for immunity when studying infections; if we had assayed survival alone we would have been led to the conclusion that *CG3066* plays no role in the fly's interactions with these microbes, when in fact, the gene plays a complicated role in defense.

The last microbe we tested was S. aureus. S. aureus infected *CG3066* mutants exhibited the most striking difference in survival with approximately an 80% reduction in the mean time to death (*p* < 0.0001) (Figure 4). Our results with S. aureus differ from those reported by Leclerc et al. who reported no difference in survival between S. aureus–infected *CG3066* mutants and wild-type flies [24]. We did not measure growth in S. aureus–infected flies because S. aureus aggregates when grown in flies and this creates a lot of scatter in colony count experiments [15]. We are therefore unable to determine whether *CG3066* mutants die from a S. aureus infection because of defects in resistance and/or tolerance properties and cannot place it in one of our predicted classes.

### CG3066 Affects the Response to Wounding

We noted that medium-injected *CG3066* mutant flies died faster than similarly treated wild-type flies. This result was missed in past publications because the survival curves were not extended until these control flies died, or these controls were apparently not performed [25,26]. To determine whether this was an effect of wounding on survival of *CG3066* mutants or whether these mutant flies were merely shorter lived, we performed lifespan analysis on unmanipulated mutant and wild-type flies. We found that the unmanipulated flies had similar life spans (Figure 4). This suggests that *CG3066* is important for tolerating some of the pathology of the wounding response.

## Discussion

By testing a panel of bacteria that cause different types of infections, we demonstrated that melanization is activated during infection, and that the degree of activation is dependent on the type of infection. We predict that there are nine potential ways of affecting resistance and tolerance of a host (Figure 1). We found that by mutating a single gene we could alter both the resistance and tolerance properties of the fly and observed up to five of the nine predicted phenotypic classes. Though not all nine possible classes were seen, we did observe the four major changes that we predicted; both tolerance and resistance could be increased or decreased by a single mutation, and these properties were dependent upon the particular microbial challenge.

The phenotypes found in *CG3066* mutant flies were somewhat surprising. We anticipated that this protease mutant would be less able to kill some bacteria and thus would show decreased resistance. Likewise we predicted that melanization might cause collateral damage and nonmelanizing flies would show increased tolerance. It was a surprise to find that melanizing mutants are more resistant to some microbes. We propose that the microbe may benefit from the damage done by the reactive oxygen because of autoimmune damage to the host; or perhaps when flies lack melanization, other, more effective immune responses show increased activity. It was also a surprise that tolerance would decrease in nonmelanizing flies. We propose that regulation could increase the activity of alternate immunity pathways that cause increased collateral damage or that the bacteria cause a different type of pathology in nonmelanizing flies that is more damaging to the host than we see in wild-type flies.

Of the nine proposed phenotypic classes (Figure 6), three should be easy to distinguish; these are cases in which resistance remains unchanged (and thus microbe levels are the same as in wild-type flies) and tolerance varies (Figure 6, right). We saw one of these classes: *CG3066* mutant flies infected with E. coli show no change in either resistance or tolerance. We interpret this as meaning that CG3066 has no effect at all on this type of infection. We did not see an example of the second or third class of mutant with our panel of bacteria. The second class would show no change in resistance but an increase in tolerance. The third would have no resistance effect and would reduce tolerance. We note that we have previously identified mutations in the second and third class. We have reported that the fly tumor necrosis factor–related molecule, eiger, is important for fighting infections with extracellular pathogens, and *eiger* mutants have decreased resistance during these types of infections. Yet during *Salmonella* infection we found that *eiger* mutants exhibit similar bacterial burdens to wild-type flies but have an extended life span, indicating that during this type of infection there is an increase in tolerance with no change in resistance. *Eiger* mutants have a balanced increase in resistance and a decrease in tolerance during a *Listeria* infection, similarly to what is seen in *CG3066* mutant flies infected with E. faecalis. *Eiger* does not appear to exhibit as many phenotypic combinations of resistance and tolerance as we observe with *CG3066* but it offers additional support that a single gene can affect both resistance and tolerance in various ways depending on the microbe [15,16]. In a published genetic screen we identified six mutants, all of which were sensitive to *Listeria* but exhibited levels of bacteria comparable to that found in wild-type flies [17]. These six mutants represent an additional phenotypic class, a decrease in tolerance with no change in resistance. It is not known if these genes can affect resistance and tolerance in additional ways with different types of infections.

**Figure 6:**
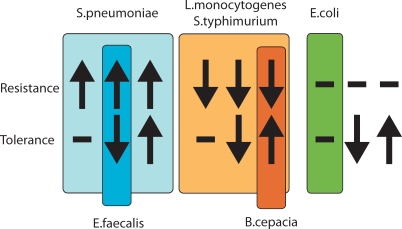
Summary of Mutant Classes We propose that resistance and tolerance can vary independently of each other, resulting in nine phenotypic classes. Three of these classes are clearly observable in *CG3066* mutants when infected with E. faecalis, B. cepacia, or E. coli. Infections of *CG3066* mutants fall into the class where resistance is increased but changes in tolerance cannot be easily measured here. Likewise, L. monocytogenes and S. typhimurium fall into the class where resistance is decreased but tolerance cannot be measured.

Three classes of phenotypes will show a decrease in resistance (Figure 6, middle). This is the phenotype we observed with L. monocytogenes and S. typhimurium infections. These bacteria grow faster in *CG3066* mutants and the flies die faster. Typically, we cannot distinguish changes in tolerance here because we do not have a good method of keeping the bacterial levels constant or correlating bacterial load and morbidity. B. cepacia provides a special case where *CG3066* mutant flies show no change in survival but show a significant increase in bacterial load when infected with this bacterium. This suggests that there must be a balanced change in resistance and tolerance in these flies.

Because we did not determine the growth of S. aureus in *CG3066* mutants, we cannot determine whether resistance and/or tolerance is affected with this microbe. If we were to consider the survival and melanization phenotypes in S. aureus-infected *CG3066* mutants only, we would suggest that it falls into the same class as L. monocytogenes and S. typhimurium. Yet, because we have seen that survival is not an accurate predicator of bacterial loads we cannot make this claim.

Another three classes of phenotypes are expected to show an increase in resistance (Figure 6, left). We saw at least one of these classes. *CG3066* mutant flies live longer than do wild-type flies infected with S. pneumoniae and are better at clearing the infection because they have a heightened resistance response. In this case we suggest that when melanization occurs, flies are less able to defend themselves against the infection; perhaps the flies waste energy on a nonproductive immune response or suffer from autoimmune damage. *CG3066* flies infected with E. faecalis provides a second special case, where we can determine changes in resistance and tolerance; the survival rate of *CG3066* mutants and wild-type parental strains were the same. This means that resistance and tolerance changes must be balanced, and we conclude that since resistance is increased in these flies, tolerance must have decreased.

We showed that CG3066 is important in controlling the nature of the infection. In the case of L. monocytogenes, we found that there are more extracellular bacteria present in *CG3066* mutants while the number of intracellular microbes remains constant. We suggest two mechanistic explanations for this change in the nature of the infection. First, melanization may be responsible for killing extracellular L. monocytogenes, and a loss in CG3066 results in an increase in extracellular bacteria. Second, CG3066 might be responsible for killing fly cells infected with L. monocytogenes, and if this does not happen, the cells may release larger numbers of bacteria into the circulation.

We made assumptions about the shape of tolerance curves when interpreting our data. We determined the life span of uninfected flies and compared this to the lifespan of infected flies and measured their bacterial levels 24 h postinfection. If these data were graphed, they would define two points and a tolerance curve could be interpolated between them. We interpreted our results as if the tolerance curve was a straight line and that each additional microbe would incrementally result in a decrease in survival. This is an assumption and should apply to some infections; however, it is possible to imagine alternative tolerance curves. We made this assumption because we do not know the actual shape of any of these curves and chose the simplest possibility. This raises the point that to truly understand the nature of microbial pathogenesis we will have to precisely define tolerance curves.

Resistance and tolerance are predicted to have very different evolutionary outcomes [37]. For resistance, if the benefits of the trait outweigh the cost of the trait then the number of hosts with that resistant trait will become more frequent in a population. As the trait occurs in higher proportions the occurrence of disease will decrease. Eventually the occurrence will become so low that the cost of the trait then outweighs the benefits of the trait and the trait will cease spreading through a population. Therefore a resistance trait is not predicted to become fixed in a population. The dynamics of a tolerance trait should be quite different. As a tolerance trait spreads through a population the occurrence of disease may also increase because more tolerant hosts are available to infect. Because the incidence of disease remains high the benefits of carrying the tolerance trait will always outweigh the costs of having it, so the tolerance trait is predicted to become fixed in a population. Our results have very important implications for the evolutionary dynamics of tolerance traits. We show that a tolerance trait can actually be quite dynamic and predict that it will not reach fixation because the same trait can serve as a resistant trait for a different pathogen. Furthermore, resistant traits are typically highly dynamic because of the coevolutionary relationship they have with pathogens, and this will feed into the system with corresponding changes in tolerance. Our findings suggest that the evolutionary dynamics of resistance and tolerance can be highly fluid even in the absence of pathogens driving such genetic instability in a host.

We noted an interaction between wounding, survival, and melanization; *CG3066* mutant flies that were given an injection of sterile medium were shorter lived compared to wild-type flies given the same treatment. Unmanipulated *CG3066* and isogenic parental lines showed no difference in survival. We have always found that medium-injected flies die faster than unmanipulated flies and do not know the pathology behind this early death. It remains possible that these flies are suffering from a cryptic infection of the native microbiota in the fly vial or that the wound healing process itself causes some pathology. These results demonstrate that *CG3066* contributes to a fly's ability to withstand this pathology.

An issue that arises as a result of the difference in the survival rate of *CG3066* flies in medium-injected flies versus unmanipulated controls is that if the medium-injected *CG3066* flies die faster than do wild-type flies, how can we determine which flies have changes in immunity? We have two answers to this question: The first is that in the three cases where we see *CG3066* flies dying faster than wild-type flies during an infection, we found that this is associated with an increased growth of bacteria and conclude that these flies have a resistance defect, in addition to other problems that they might have. The second answer is that we suggest the flies are dying for different reasons in medium-injected versus pathogen-infected flies, and that the two processes do not necessarily correlate with each other. We do not know the cause of pathology in either death by infection or death by wounding and have no reason to suspect that they are identical.

Recent studies suggested that these immune mechanisms are dispensable in *Drosophila* with respect to their importance for survival to microbial infections or, at best, serve a redundant role [24,25]. These experiments were carried out by assaying the phenotypes of *CG3066* mutants. We are careful to limit the analysis of our phenotypes to the effects of CG3066 and do not go so far as to state that the loss of melanization is the cause for the phenotypes we observe. It remains possible that CG3066 serves additional roles in fly immunity and does not solely activate phenoloxidase. Here we report that the response initiated by CG3066 is important for surviving infections and that its effects are dependent on the type of infection. This response affects both resistance and tolerance mechanisms in *Drosophila*. We suggest that the importance of these mechanisms was missed previously because past studies utilized microbes that do not induce a strong melanization response in the fly, did not measure bacterial loads in the infected flies, did not take the survival curves out to completion, and compared mutants to other mutants instead of to wild-type flies.

The findings reported here have implications about the evolution of immunity; they suggest that polymorphisms that increase resistance to one pathogen may reduce the resistance or tolerance to other pathogens and thus the defense response of a given fly strain will likely be optimized by its interaction with microbes in the wild. As a result, there is likely no “best” solution that produces a perfect immune system, only an equilibrium that allows the fly to survive the pathogenic threats that its ancestors have faced. This equilibrium will require the balancing of both resistance and tolerance, and thus we can not completely understand the defensive properties of a host unless we measure both of these properties in response to a variety of pathogens.

## Materials and Methods

### Fly strains.

The wild-type parental strain used in all experiments is *white^1118^* (Bloomington stock center, stock 6326) The *CG3066 ^KG02818^* allele was obtained from Bloomington stock center and backcrossed onto the *white^1118^* background for four generations. Flies were kept in standard fly bottles containing dextrose medium.

### Bacterial strains.

All strains used are listed in Table 1.

### Pathogen culture conditions.

S. pneumoniae cultures were grown standing at 37 °C 5% CO_2_ in brain heart infusion medium (BHI) to an OD_600_ of 0.15 and aliquots were frozen at −80 °C in 10% glycerol. For infection, an aliquot of S. pneumoniae was thawed and diluted 1:3 with fresh BHI medium and allowed to grow to OD_600_ of 0.15 at 37 °C 5% CO_2_. Bacteria was then concentrated to an OD_600_ of 0.3 in PBS. E. coli, E. faecalis, and S. typhimurium cultures were grown overnight at 37 °C in Luria Bertani (LB) medium. E. coli and E. faecalis cultures were shaken, while S. typhimurium cultures were grown standing. E. coli and S. typhimurium cultures were diluted to OD_600_ of 0.1 with fresh LB medium prior to injection. E. faecalis cultures were diluted to an OD_600_ of 0.05 with medium. B. cepacia cultures were grown standing overnight in LB medium at 29 °C and injected at an OD_600_ of 0.001. L. monocytogenes and S. aureus were grown in BHI medium. L. monocytogenes was grown standing and injected at an OD_600_ of 0.01. S. aureus was grown shaken and injected at an OD_600_ 0.001.

### Injections.

Male 5- to 7-d-old files were used for injection. Flies were anesthetized with CO_2_ and injected with 50 nl of culture using a picospritzer and pulled glass needle. Flies were injected in the anterior abdomen on the ventrolateral surface. Flies were then placed in vials containing dextrose medium in groups of twenty and incubated at 29 °C, 65% humidity with the exception of B. cepacia, which was incubated at 18 °C with no humidity control.

### Survival curves.

For each microbe tested, *w^1118^* and *CG3066* mutants were injected with the microbe or medium as a control. Flies were placed in dextrose vials in groups of 20 after injection and a total of sixty flies were assayed for each condition. The number of dead flies was counted daily. Using Prism software, Kaplan-Meier survival curves were generated and statistical analysis was done using log-rank analysis. Survival was tested for each microbe listed in Table 1 at least three times and gave similar results for each trial.

### CFU determination and gentamicin chase.

Infected flies were homogenized in media supplemented with 1% Triton X-100 and serially diluted. S. pneumoniae-infected flies were homogenized in PBS without triton. Dilutions were plated on LB agar plates (blood agar plates for S. pneumoniae) and incubated overnight. The data was plotted as box-and-whisker plots using Graphpad Prism software for three independent experiments. The *p*-value was determined with a nonparametric two-tailed *t*-test. For the gentamicin chase experiments, flies were injected with 50 nl of 1 mg/ml gentamicin or water 3 h prior to homogenizing and plating.

## Abbreviations

PPO: prophenoloxidase
